# NO^●^ Represses the Oxygenation of Arachidonoyl PE by 15LOX/PEBP1: Mechanism and Role in Ferroptosis

**DOI:** 10.3390/ijms22105253

**Published:** 2021-05-17

**Authors:** Karolina Mikulska-Ruminska, Tamil S. Anthonymuthu, Anastasia Levkina, Indira H. Shrivastava, Alexandr A. Kapralov, Hülya Bayır, Valerian E. Kagan, Ivet Bahar

**Affiliations:** 1 Department of Computational and Systems Biology, School of Medicine, University of Pittsburgh, Pittsburgh, PA 15260, USA; ihs2@pitt.edu; 2 Institute of Physics, Faculty of Physics, Astronomy and Informatics, Nicolaus Copernicus University in Toruń, Grudziadzka 5, 87-100 Toruń, Poland; 3 Department of Critical Care Medicine, Safar Center for Resuscitation Research, Children’s Neuroscience Institute, Children’s Hospital of Pittsburgh, University of Pittsburgh, Pittsburgh, PA 15260, USA; ATAMIL@pitt.edu (T.S.A.); bayihx@ccm.upmc.edu (H.B.); 4 Department of Environmental and Occupational Health and Center for Free Radical and Antioxidant Health, University of Pittsburgh, Pittsburgh, PA 15260, USA; levkina.a.anastasia@gmail.com (A.L.); olk6@pitt.edu (A.A.K.); 5 Institute of Translational Medicine, Pirogov Russian National Research Medical University, Ostrovityanova 1, 117997 Moscow, Russia; 6 Department of Radiation Oncology, University of Pittsburgh, Pittsburgh, PA 15260, USA; 7 Department of Chemistry, University of Pittsburgh, Pittsburgh, PA 15260, USA; 8 Department of Pharmacology and Chemical Biology, University of Pittsburgh, Pittsburgh, PA 15260, USA; 9 Institute of Regenerative Medicine, IM Sechenov Moscow State Medical University, 119048 Moscow, Russia

**Keywords:** nitric oxide, ferroptosis, lipid peroxidation, lipoxygenase structure, O_2_ and NO^●^ binding mechanisms, 1-stearoyl-2-arachidonoyl phosphatidylethanolamine (1-SA-2-ETE-PE or SAPE), lipidomics, MD simulations

## Abstract

We recently discovered an anti-ferroptotic mechanism inherent to M1 macrophages whereby high levels of NO^●^ suppressed ferroptosis via inhibition of hydroperoxy-eicosatetraenoyl-phosphatidylethanolamine (HpETE-PE) production by 15-lipoxygenase (15LOX) complexed with PE-binding protein 1 (PEBP1). However, the mechanism of NO^●^ interference with 15LOX/PEBP1 activity remained unclear. Here, we use a biochemical model of recombinant 15LOX-2 complexed with PEBP1, LC-MS redox lipidomics, and structure-based modeling and simulations to uncover the mechanism through which NO^●^ suppresses ETE-PE oxidation. Our study reveals that O_2_ and NO^●^ use the same entry pores and channels connecting to 15LOX-2 catalytic site, resulting in a competition for the catalytic site. We identified residues that direct O_2_ and NO^●^ to the catalytic site, as well as those stabilizing the esterified ETE-PE phospholipid tail. The functional significance of these residues is supported by in silico saturation mutagenesis. We detected nitrosylated PE species in a biochemical system consisting of 15LOX-2/PEBP1 and NO^●^ donor and in RAW264.7 M2 macrophages treated with ferroptosis-inducer RSL3 in the presence of NO^●^, in further support of the ability of NO^●^ to diffuse to, and react at, the 15LOX-2 catalytic site. The results provide first insights into the molecular mechanism of repression of the ferroptotic Hp-ETE-PE production by NO^●^.

## 1. Introduction

Recent years have brought attention to ferroptosis, an iron-dependent form of regulated cell death implicated in a broad range of diseases [1,2], and to the selective peroxidation of eicosatetraenoyl-phosphatidylethanolamine (ETE-PE) by 15-lipoxygenase (15LOX) complexed with PE-binding protein 1 (PEBP1) [3,4,5] as a ferroptotic mechanism. ETE-PE peroxidation produces hydroperoxy-ETE-PE (HpETE-PE) molecules that, in turn, serve as pro-ferroptotic signals. 15LOX catalytic action includes the Fe-driven abstraction of a hydrogen atom from the carbon in bis-allylic position, formation of a carbon-centered radical followed by addition of molecular oxygen, culminating in the production of 15-HpETE.

The role of 15LOX in the onset of ferroptosis is now well-established [3,4,5,6]. Under physiological conditions, arachidonic acid (AA), a polyunsaturated fatty acid (PUFA) also termed *cis*-5,8,11,14-ETE or shortly ETE, is the preferred substrate of these non-heme iron-containing enzymes; but under inflammatory conditions, such as those stimulated by interleukin-13/4, the formation of the complex 15LOX/PEBP1 shifts the substrate-specificity of 15LOX from free AA to AA esterified into PE (or ETE-PE). The AA-esterification of PE mostly occurs at the sn-2 acyl chain leaving the sn-1 chain for saturated or mono-unsaturated acyls, predominantly palmitic (C16:0), stearic (S) (C18:0), and oleic (C18:1). Of interest below will be 1-SA-2-ETE-PE, i.e., PE with stearic acid (SA) and arachidonic acid (AA or ETE) at the respective sn-1 and sn-2 chains, which will be shortly called SAPE. 15LOX-catalyzed peroxidation of SAPE at its sn-2 chain results in the production of 15-HpHETE-PE [3]—one of the major players in the ensuing induction of ferroptosis. As such, the 15LOX-2/PEBP1 complex emerged as a master promoter of ferroptotic cell-death [3]. This involvement of 15LOX-2/PEBP1 has been supported by the ability of ferrostatin-1, the most common ferroptosis inhibitor, to bind the complex [4] and inhibit the formation of 15-HpHETE-PE.

As a multistage process, ferroptosis is regulated by several enzymatic events occurring independently or concertedly: (i) biosynthesis of PUFA-PE peroxidation substrates by acyl Co-A synthetase 4 (ACSL4) and lysophosphatidylcholine acyltransferase (LPCAT) [7]; (ii) prevention of the formation of 15-HpETE-PE by flavin-containing oxidoreductase, ferroptosis suppressor protein 1 (FSP1) [8,9]; and (iii) reduction in 15-HpETE-PE into an innocuous alcohol, 15-HETE-PE, by glutathione peroxidase 4 (GPX4) [10,11]. Lately, this list of ferroptosis regulators has been extended to include new members, such as the Ca^2+^-independent phospholipase A2β (iPLA2β) [12], which can hydrolyze peroxidized phospholipids and, therefore, can eliminate the ferroptotic cell death signal, and the inducible isoform of nitric oxide synthase, iNOS, that generates NO^●^ [13]. Notably, NO^●^’s reactivity towards different radical intermediates of lipid peroxidation has been discovered more than three decades ago [14]. Furthermore, NO^●^ inhibits lipid peroxidation by lipoxygenase and cyclooxygenase [15].

In the present study, we first performed a lipidomics analysis to observe that NO^●^ indeed suppresses the production of HpETE-PE by 15LOX-2/PEBP1, while promoting the formation of nitrosylated ETE-PE. As the 15LOX-2/PEBP1 complex, rather than 15LOX-2 alone, catalyzes the oxidation of 15-ETE-PE (rather than free ETE), we focused here on the interactions of NO^●^ with SAPE and15LOX-2/PEBP1, and on its competition with the lipoxygenase substrate O_2_. Notably, the precise delivery of molecular oxygen to form a peroxyl radical from the carbon-centered radical of ETE at the 15LOX-2 catalytic site occurs via a specialized channel, whose structure and accessibility is altered upon binding of ETE to the enzyme [16]. With this in mind, we studied the effects of NO^●^ peroxidation of SAPE by 15LOX-2/PEBP1 and the structural dynamics of the oxygen delivery process to the catalytic Fe^3+^ in the 15LOX-2/PEBP1 complex and the competition with NO^●^, in the presence and absence of the ETE-PE substrate compared to free ETE in 15LOX-2. Our study provides a mechanistic description of the time-evolved interactions between 15LOX-2 (bound to PEBP1) and its ligands, the ETE-PE- and PEBP1-induced changes in 15LOX-2 conformation or catalytic site accessibility which alter the delivery of O_2_ and NO^●^, and the competition between O_2_ and NO^●^ for binding the active site. Several key residues mediating the events prior to peroxidation and the interference of NO^●^ to repress them are identified, the critical roles of which are consolidated by in silico saturation mutagenesis experiments and comparisons with recent work.

## 2. Results

### 2.1. Lipidomics Analysis of the Effect of NO^●^ on 15LOX-2/PEBP1 Peroxidation Activity

**Figure 1:**
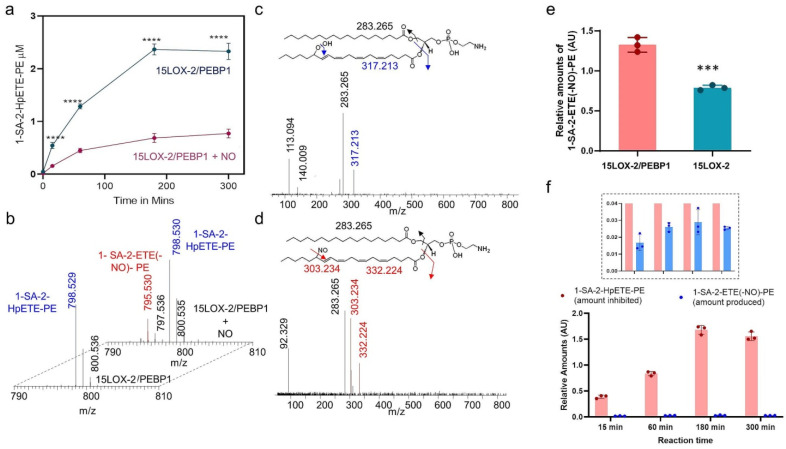
Effect of NO^●^ on peroxidation of 1-SA-2-ETE-PE by the 15LOX-2/PEBP1 complex. (**a**) Kinetics of the production of 1-SA-2-HpETE-PE by 15LOX-2/PEBP1 in the presence and absence of NO^●^ donor. Data are mean ± SD, *n* = 3, *** definition; **** *p* < 0.0001 vs. 15LOX-2/PEBP1 + NO, two way ANOVA, Sidak post-hoc analysis. (**b**) Mass spectrum showing the nitrosylated product formed upon 1-SA-2-ETE-PE incubation with 15LOX-2/PEBP1+PAPA NONOate (back). Nitroso-1-SA-2-ETE-PE (1-SA-2-ETE(-NO^●^)-PE) was not detected in the system without PAPA NONOate (front). Fragmentation analysis of 1-SA-2-HpETE-PE (**c**) and 1-SA-2-ETE(-NO)-PE (**d**) showing representative fragments. The insets at the top show the structures of 1-SA-2-HpETE-PE, 1-SA-2-ETE(-NO)-PE and their possible fragments. (**e**) Bar plot comparing 1-SA-2-ETE(-NO)-PE formation by 15LOX-2 and by 15LOX-2/PEBP1, both in the presence of NO^●^. Data are mean + SEM., *n* = 3, *** *p* = 0.0007, student’s *t*-test. (**f**) Bar plot showing that the amount of 1-SA-2-HpETE-PE inhibited by NO^●^ is significantly larger than the amount of 1-SA-2-ETE(-NO)-PE produced, as a function of reaction time. The inset bar plot shows the enlarged view of the lower plot, between the ordinate values 0.0 < y < 0.04 (AU).

To examine the effects of NO^●^ on the production of the pro-ferroptotic signal, 15-HpETE-PE, by 15LOX-2/PEBP1, we incubated one of the lipoxygenase isoforms, 15LOX-2, and PEBP1 in the presence of an NO^●^-donor, propylamine (PAPA) NONOate [17]. This donor releases NO^●^ at a constant rate with a decay half-time of 15 min. We found that NO^●^ strongly suppressed the production of hydroperoxide of 1-SA-2-ETE-PE (HpETE-PE) (Figure 1a). Detailed analysis of redox lipidomics data led to the identification of a nitrosylated product, nitroso-ETE-PE (1-SA-2-ETE(-NO)-PE) generated by 15LOX-2/PEBP1 only in the presence of NO^●^ (Figure 1b–d).

These observations suggest that a direct reaction occurs between the ETE-PE carbon-centered radical, an intermediate of the 15LOX-catalyzed dioxygenase cycle [18], and the NO^●^ radical. No nitroxygenated products formed from the reaction of O_2_ with the reactive intermediates of NO^●^ [19] could be found. Notably, the 15LOX-2/PEBP1 complex produced a greater amount of NO^●^-ETE-PE than 15LOX-2 alone (Figure 1e). Lack of appropriate standards of nitrosylated ETE-PE precluded the absolute quantification of nitrosylated ETE-PE and its comparison with 1-SA-2-HpETE-PE levels. However, upon normalization with the same internal standard, the relative amounts of the nitrosylated ETE-PE were about 50 folds lower than the inhibited amounts of 1-SA-2-HpETE-PE (Figure 1f).

Combined, these results suggest that at least two mechanisms contribute to the observed suppression of ETE-PE peroxidation by NO^●^ in the 15LOX-2/PEBP1 complex: (i) direct reaction of NO^●^ with the ETE-PE carbon-centered intermediate, and (ii) potential presence or occupancy of the O_2_-binding site and channeling path by NO^●^ or a competition between the two small molecules—O_2_ and NO^●^—interfering with the peroxidation of the oxidizable substrates. To explore the latter mechanism, we analyzed the changes in the conformational state and dynamics of 15LOX-2/PEBP1 invoked upon binding of the oxidizable substrate, as well as O_2_ and NO^●^.

### 2.2. In silico Analysis of the Effect of NO^●^ on the Structure, Dynamics, and Interactions of 15LOX-2/PEBP1

We performed structure-based modeling and simulations to investigate the molecular basis of the experimentally observed reduced activity of 15LOX-2/PEBP1 in the presence of NO^●^. First, we explored how the accessibility of the catalytic site of 15LOX-2 to O_2_ and NO^●^ molecules is altered upon complexation with PEBP1. Next, we investigated how the presence of NO^●^ affects O_2_-binding and channeling to the catalytic pocket. We further analyzed the effect of oxidizable substrates (1-SA-2-ETE-PE, shortly called SAPE, vs. free ETE (AA)) on the accessibility of the 15LOX-2 catalytic site to O_2_ and NO^●^, in the presence and absence of PEBP1. Overall, we conducted 10 sets of molecular dynamics (MD) simulations in duplicate (or triplicate) runs of 150 nanoseconds (ns) under different conditions, summing up to total simulation duration of 3.6 microseconds in explicit water, as summarized in the Supplementary Table S1.

### 2.3. Specific Porous Regions on 15LOX-2 Surface Enable Access of O_2_ and NO^●^ to the Catalytic Cavity

**Figure 2:**
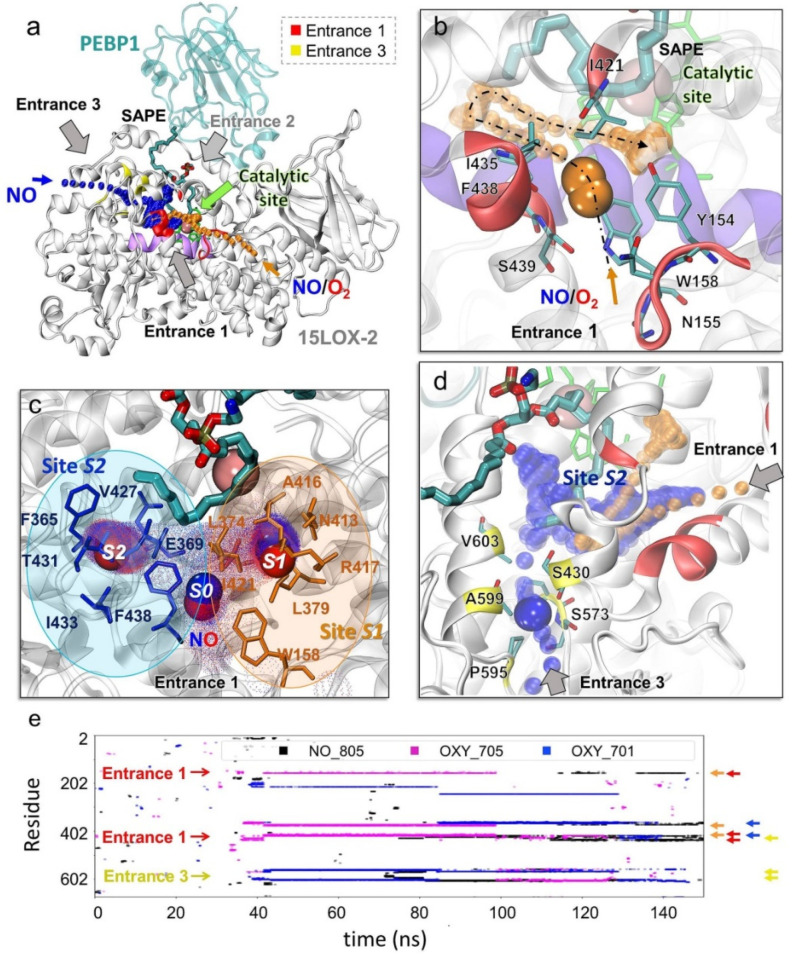
Ligand (NO^●^ and O_2_) binding and channeling to the catalytic site of 15LOX-2 and critical interactions mediating this process in the presence of SAPE. Results from MD simulations for 15LOX-2/PEBP1 in the presence of SAPE, five NO^●^ and five O_2_ molecules are presented (Supplementary Table S1; last row). (**a**) 15LOX-2/PEBP1/SAPE complex stabilized by the end of a 150 ns MD run. 15LOX-2 and PEBP1 are displayed in white and cyan ribbon diagrams, respectively; and SAPE in cyan sticks (with atoms in CPK colors). Catalytic residues (H373, H378, H553, I676) are in green sticks, and the iron ion in pink sphere. Entrances 1 (red), 2 (inaccessible in the presence of PEBP1 and substrate), and 3 (yellow) are indicated by black/gray arrows. The series of orange dots represents the entry or diffusion path of O_2_ and NO^●^ molecules through Entrance 1, and the blue dots those through Entrance 3. The region between helices α12 and α14 (K365-L380, UniProt ID: O15296), colored violet, contains two of the catalytic histidines. (**b**) A close-up view of Entrance 1, from a different angle. An O_2_ molecule (orange) approaches it around 37 ns and translocate to the catalytic site (orange dots in panels b and d) (**c**) A close-up view of the catalytic site with three NO^●^ molecules occupying the recognition site *S0* (Entrance 1), and the binding sites *S1* (orange) and *S2* (blue). The cloud of small dots shows where and how long (more dots) NO^●^ travelled or remained bound during simulations. NO^●^ molecules are in blue-red spheres corresponding to their nitrogen and oxygen atoms, respectively. (**d**) A close-up view of the Entrance 3 providing access to a NO^●^ molecule (in blue). Key residues lining the path are displayed in yellow. The series of orange dots represents the entry or diffusion path for O_2_ and NO^●^ through Entrance 1 and the blue dots show the path through Entrance 3. (**e**) Illustration of the time evolution of contacts between 15LOX-2 residues (ordinate) and O_2_/NO^●^ molecules (labeled NO_805, OXY_701 (shown in panel b) and OXY_705; shown by the respective black, magenta, and blue traces) during a typical MD run. Red, yellow, orange, and blue arrows along the ordinate correspond to the Entrance 1 (*E1*), Entrance 3 (*E3*), Binding Sites *S1* and *S2*, respectively. All three molecules were arrested for extended durations at *S1* or *S2*, inserting to the catalytic site through Entrance 1 or 3.

Lipoxygenases are non-heme iron-containing enzymes. The iron at the catalytic site in the central part of the catalytic domain of 15LOX-2 is coordinated by four highly conserved residues including three histidines: H373, H378 (on helix α12-14), H553 (on helix α27), and the C-terminal isoleucine, I676 (Supplementary Figure S1). Simulations revealed three porous regions on 15LOX-2 surface, denoted here as Entrances 1–3 (*E1–E3*), which could potentially allow for the entry of the radicals, O_2_ or NO^●^_,_ to potentially access the catalytic cavity of the enzyme (Figure 2a): *E1*, near the loop Y154-P159 where Y154 and W158 side chains appear to be gating the entrance (Figure 2b and Supplementary Figure S1a,c); *E2*, at the 15LOX-2/PEBP1 binding interface (Figure 2a); and *E3*, near S573, P595, and A599 (Figure 2d). Although all three entrances could potentially provide access to the catalytic site [13], *E2* was largely obstructed by the oxidizable substrate (SAPE or AA in our simulations) and, therefore, inaccessible to O_2_ and NO^●^. In contrast, *E1* served as the main access point to a tunnel leading to the catalytic site, for both O_2_ and NO^●^ (Figure 2a,b, orange trace), while *E3* provided an alternative path leading to the catalytic site (Figure 2a, blue trace and Figure 2d). Supplementary Figure S1 provides close-up views of *E1* and *E3* from different perspectives, to facilitate the visualization of their connection to the catalytic site; and Supplementary Figure S2 describes the secondary structure of 15LOX-2 [20].

The entrance *E1* was lined by Y154, N155, G157, W158, I421, I435, F438, and S439 (Figure 2b). It was transiently occupied by NO^●^ or O_2_ at the initial stage of their interaction with the enzyme. After this first recognition event (at site *S0*), the small molecules moved deeper towards two attractive sites, binding sites *S1* and *S2*, that arrested them for extended durations (Figure 2c): *S1*, composed of N413, A416, R417, L374, and L379, was occupied by these ligands for more than 70 ns; and *S2*, containing I433, T431, V427, F365, and E369, for more than 35 ns, during 150 ns runs. Both sites made close contacts (atom-atom distance less than 3.5 Å) with NO^●^ or O_2_ for sufficiently long durations (Figure 2e) meeting the criteria (see Methods) to qualify as binding sites.

The entrance *E3*, on the other hand, included (in addition to S573, P595, and A599), S430 and V603 near the surface (Figure 2d). These residues assist in directing the O_2_/NO^●^ molecules from *E3* to the catalytic site. Entrances 1 and 2 are predominantly composed of hydrophobic residues: (i) the cluster I216, I604, F561, C564, and A565 that initially bound O_2_/NO^●^ for ~40 ns; and (ii) the cluster L607, L610, L420, V426, and V427 that retained the molecules for ~11 ns. The third site that serves as a bridge between *E3* and catalytic site partially overlaps with *S2*: it contains the residues L246, E364, F365, H368, E369, and L570 that bound O_2_/NO^●^ for ~40 ns. Thus, *S2* serves as an attractor for O_2_ or NO^●^ binding entering through either *E1* or *E3*. Two highly conserved residues therein, F365 and E369, will be shown below to play an important role in binding or redirecting the O_2_ and NO^●^ molecules.

A summary of all these residues involved in various roles, entry (*E1* and *E3*), binding to *S1* and *S2*, or insertion towards the catalytic site (mainly hydrophobic clusters), is given in Supplementary Table S2, along with corresponding secondary structures (based on [21]).

These simulations also indicated an additional pore that connected from the surface to the catalytic site through a short tunnel with a wide entrance. This path, available in both unbound- and PEBP1-bound-15LOX-2 (Supplementary Figure S3a,b, in green), was not selected by either NO^●^ or O_2_. This tunnel included five residues highly conserved across LOX family members [22], L610, Q560, and the three catalytic residues, H373, H553, I676, along with E613 and S557. It is conceivable that this tunnel plays a role in 15LOX-2 dioxygenase activity, yet to be explored.

### 2.4. O_2_ and NO^●^ Compete for the Catalytic Site

**Figure 3:**
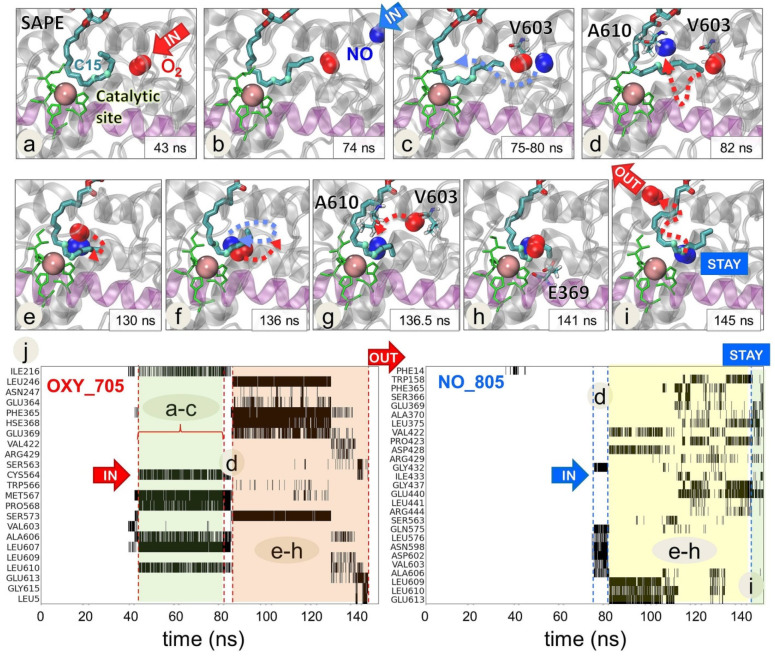
Competition between NO^●^ and O_2_ molecules near the C15 atom of the AA-chain of SAPE at the catalytic site of 15LOX-2 complexed with PEBP1. A sequence of MD snapshots (**a**–**i**) illustrates the time evolution of the positions of a NO^●^ radical (NO_805) and an O_2_ molecule (OXY_705) during the period 43 < t < 150 ns of the simulations. A first O_2_ (**a**) reaches the catalytic site at t = 43 ns, followed by a NO^●^ at t = 74 ns (**b**,**c**) which disrupts the binding of O_2_. After a competition process during which the two molecules undergo multiple fluctuations and dislocations in their positions, the O_2_ molecule ends up leaving the catalytic cavity at 145 ns (**i**). Catalytic site residues (H373, H378, H553, and I676) are displayed in green and sn-2 chain of SAPE as cyan-red-blue sticks. Two highlighted atoms of SAPE (light cyan spheres) denote C13 and C15 atoms as reference points. NO^●^ and O_2_ are shown in blue and red spheres, respectively. The region between the helices α12 and α14 is colored in light violet. Lower panels (**j**) display the detailed time evolution of contacts between 15LOX-2 residues and O_2_ (**left panel**) and NO^●^ (**right panel**) molecules. This panel includes only those residues that undergo frequent contacts (cumulative contact time of 2.5 ns) with O_2_ (**left**) or NO^●^ (**right**) during the simulation period of 150 ns. Contacts are shown by black shades/bars for each residue. A603 and A606 are involved in initial contacts with O_2_ which are broken upon the arrival and interference of NO^●^. See Movie 1 for a visualization of the interplay between O_2_ and NO^●^ at the catalytic site.

Simulations with the substrate revealed an interesting effect which arises upon the complexation 15LOX-2 with PEBP1, namely a competition between O_2_ and NO^●^ for a position near the C15 carbon in the arachidonoyl chain of SAPE within the catalytic site (Figure 3, Movie 1). It is known that 15LOX-2/PEBP1 complex converts SAPE substrate to its peroxidized form by facilitating the addition of a hydroperoxyl- group at the C15 position of the ETE sn-2 chain at the catalytic site. Thus, the presence of an O_2_ molecule close to C15 is necessary to initiate the process. Here, we observed that the positioning of O_2_ at C15 was disrupted by the interference of NO^●^ radicals. Figure 3 illustrates such an observation. Panels a–i display a series of snapshots showing how NO^●^ approaches the region originally occupied by an O_2_ molecule, to displace O_2_, and position itself near the C15 atom for extended durations (>50 ns), until the departure of O_2_ from the catalytic site, consistent with the experimentally observed ability of NO^●^ to inhibit the peroxidation of SAPE.

The panel j in Figure 3 indicates the 15LOX-2 residues (ordinate) that make successive contacts with NO^●^ and O_2_ molecules, starting from first encounters with O_2_ (left panel, 40 < t < 75 ns, shaded in green), followed by the arrival of NO^●^ to the catalytic site and the competition between NO^●^ and O_2_ for binding the catalytic pocket residues (both panels, 80 < t < 140 ns; shaded in orange (left) and yellow (right)), finally ending with the dislocation of O_2_, while NO^●^ remained stabilized near C15 and Fe^3+^ ion. Among residues ligating (successively or competitively) both O_2_ and NO^●^ during this trajectory, we note the highly conserved residues P365 and E369 in site S2, and the hydrophobic cluster V603, A606, L609, and L610, revealing that O_2_ and NO^●^ channeled to the catalytic site through Entrance 3 in this case.

### 2.5. Selected Residues Stabilize O_2_ and NO^●^ Near 15LOX-2 Catalytic Site in a Substrate-Dependent Manner

We further carried out a statistical analysis of four independent runs to identify the 15LOX-2 residues which made frequent and long-lived contacts with O_2_/NO^●^ molecules (see Methods), among those located within 20 Å of the Fe^3+^ ion. Supplementary Figure S4a displays such residues. Panels b–d list the PEBP1-bound 15LOX-2, free 15LOX-2 and PEBP1 residues, respectively, that make contacts for extended durations with O_2_ and NO^●^, and the corresponding number of runs in which such contacts are observed. Clearly 15LOX-2 residues at Entrances 1 and 3 (*E1* and *E3*) are detected among them, along with sites *S1* and *S2* residues, as indicated by the bars between panels b and c. Of particular importance is the long helix α12-14 (F365-L379) comprising both *S1* (L374, L379) and *S2* (F365, E369) residues also noted [13] in the absence of the phospholipid substrate. Interestingly, our recent computational analysis of a dataset of 88 crystal structures resolved for lipoxygenase showed that this particular region acts as a strong effector of allosteric signals [22]. The highly conserved WxxAK motif (W353-K357) shared by other LOXs [22] also takes part in the same region. Comparison of panels b and c shows that certain residues (E168, L172-A179 (except for I174), and F219-P223) interact with O_2_ and NO^●^ in the absence of PEBP1 but become inaccessible upon PEBP1 binding.

### 2.6. The Change in 15LOX-2 Structure Upon PEBP1 Binding Renders the Catalytic Site Accessible to Both O_2_ and NO^●^ Molecules

**Figure 4:**
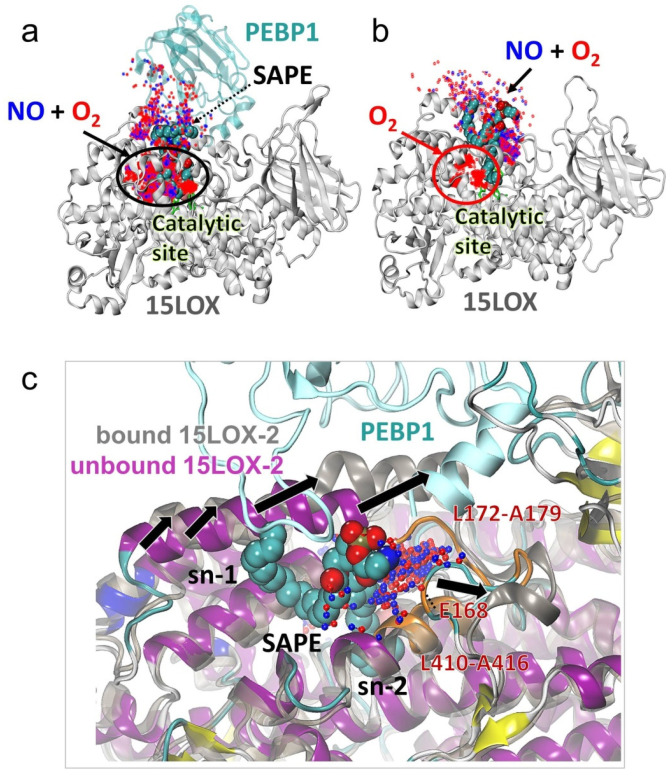
Conformational change in 15LOX-2 induced upon PEBP1 binding allows NO^●^ binding to the catalytic sites in the presence of SAPE. Panels **a** and **b** compare the binding patterns of O_2_/NO^●^ to 15LOX-2 in the presence (**a**) and absence (**b**) of complexation with PEBP1. The positions of NO^●^ (blue dots) and O_2_ (red dots) sampled during MD snapshots are displayed. These refer to contacts (within 3.5 Å) between O_2_/NO^●^ and (**a**) SAPE-bound 15LOX-2/PEBP1 complex and (**b**) SAPE-bound 15LOX-2 (with O_2_/NO^●^ molecules within 7 Å from any SAPE atom). Both O_2_ and NO^●^ molecules sample the catalytic site in the presence of PEBP1 (panel a, black oval). In the absence of PEBP1 the catalytic site exclusively harbors the O_2_ molecules (red oval). Snapshots from another run (Supplementary Figure S5) illustrate the reproducibility of the results. (**c**) Structural change induced by PEBP1 binding. Structural alignment of 15LOX-2 structure (after 150 ns simulation) in PEBP1-bound (dark grey) and unbound (magenta) forms with SAPE substrate (spheres) and accumulation of NO^●^ molecules near the loop L172-A179 (orange) on the surface, are shown. Black arrows show the direction of conformational change in the α2 helix and the T166-A179 loop, providing access to both NO^●^ (blue dots) and O_2_ (red dots). Other NO^●^ molecules attracted by the stearic acid sn-1 chain of SAPE are hidden for better visualization.

We observed that in the presence of PEBP1, both O_2_ and NO^●^ co-localize within the catalytic site (Figure 4a) where they compete for a position near the C15 atom of SAPE (Figure 3, Movie 1). Remarkably, in the absence of PEBP1 and at the small concentration of NO^●^, the NO^●^ molecules were unable to access the catalytic site (Figure 4b, red oval). This behavior consistently reproduced in independent runs (see Supplementary Figure S5) was due to a conformational change (opening or exposure of a binding site) between the α2 helix and the T166-A179 region stabilized upon PEBP1 binding (black arrows Figure 4c). When this site was not exposed (in the absence of PEBP1), O_2_ and NO^●^ molecules were attracted to the hydrophobic sn-1 (stearoyl) chain of SAPE and to the 15LOX-2 residues L172-A179. At a higher NO^●^ concentration, however, this effect was partially suppressed and a few NO^●^ molecules took a place near C15 atom of SAPE in the catalytic site (black dotted oval Supplementary Figure S6). Simulations repeated with AA bound to 15LOX-2 (Supplementary Figure S7) showed that NO^●^ molecules were able to access the catalytic site in the absence of the sn-1 chain of SAPE that otherwise sequestered the NO^●^ molecules.

These simulations therefore lead to the following conclusions: (i) PEBP1 increases the affinity of 15LOX-2 to bind both O_2_ and NO^●^ molecules to the catalytic site; whereas in the absence of PEBP1 and without higher NO^●^ concentration, only O_2_ molecules access the active site, while NO^●^ molecules preferentially bind to the phospholipid tail of SAPE; (ii) competitive binding of NO^●^ to the catalytic site in the presence of PEBP1 (Figure 4a) is expected to interfere with the peroxidation activity of 15LOX-2 resulting in lipid nitrosylation, as well as decreased conversion of SAPE to SAPE-OOH; and (iii) in absence of PEBP1, the NO^●^ molecules co-localize at the exposed sn-1 stearoyl chain and do not compete with the O_2_ molecules at the catalytic site (Supplementary Figure S5b).

### 2.7. The Precise Positioning of SAPE for Peroxidation by 15LOX-2 Is Assisted by α2 Residues N181, Y185, and G189, and by E3/S1 Residues A416 and A606

**Figure 5:**
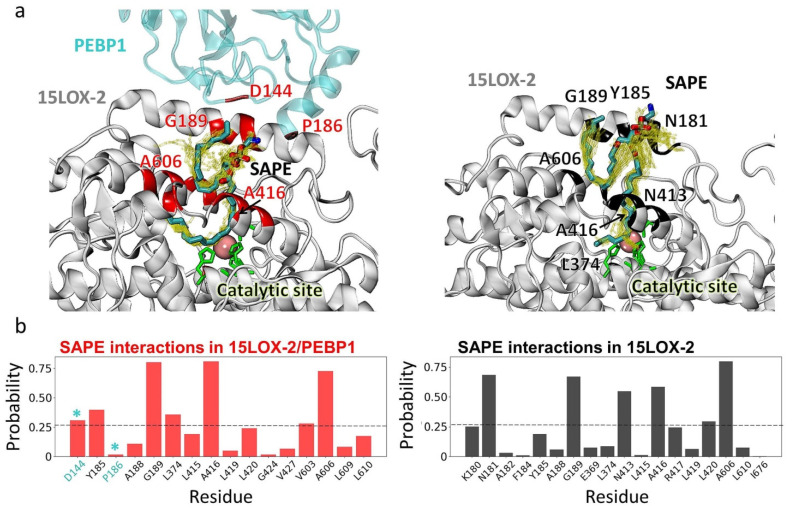
Substrate-binding residues of 15LOX-2 in the presence and absence of PEBP1. (**a**) Interfacial contacts (within 3.5 Å) between the substrate SAPE and PEBP1/15LOX-2 (left panel) and 15LOX-2 (right panel) observed in MD simulations. (**b**) 15LOX-2 residues exhibiting the highest probabilities of contacts with SAPE. The ordinate shows the probabilistic occurrence (the number of counts divided by the total number of selected frames that satisfy the contact requirement). Two PEBP1 residues, D144 and P186, also observed to make frequent contacts are also included (indicated by cyan stars). The fluctuations in the conformation of SAPE during simulations are indicated by yellow sticks.

Lipid peroxidation by 15LOX-2 requires O_2_ molecule to be positioned in proximity (<7.5 Å) of the carbon C15 in the arachidonoyl chain of SAPE, while the C13 in the arachidonoyl chain of SAPE would approach (by <7.5 Å) the iron ion at the catalytic site. We analyzed the MD trajectories to examine whether such poses were sampled. We identified a total of 121 and 538 poses, respectively, for PEBP1-bound and -unbound 15LOX-2/SAPE (Figure 5a) that satisfied this requirement. The 15LOX-2 residues, G189, A416 (at *S1*) and A606 (at *E3*), were distinguished by their high tendency (75% of MD snapshots) to coordinate SAPE, in both systems. Furthermore, α2-helix residues N181, Y185, and G189 exhibited a high propensity to coordinate the substrate, Y185 playing a major role in the presence of PEBP1; and N181 in the absence. Additionally, PEBP1 D144 and P186 contributed to stabilizing the SAPE.

### 2.8. In Silico Saturation Mutagenesis Analysis Confirms the Critical Role of Selected Residues Amongst Those Identified to Mediate O_2_/NO^●^ Entry and Translocation to the Catalytic Site

The present study points to several residues implicated in initial entry, channeling, and binding of O_2_/NO^●^ taking part in *E1* or *E3*, or associated sites *S1, S2*, and other clusters (of mostly hydrophobic residues) paving the way to the catalytic site (Table S2). As a test of the potential impact of substitutions at those sites, we performed an in silico saturation mutagenesis analysis, using *Rhapsody* [23]. This machine learning tool scans all possible 19 substitutions at all the *N* amino acid positions of the protein to predict the so-called pathogenicity probabilities, a measure of the expected impact of specific substitutions on the protein function, varying from zero (neutral) to 1 (most damaging or pathogenic). The predictions are based on the evaluation of the sequence-, structure- and dynamics-properties of 15LOX family proteins in comparison to the features observed in more than 20,000 missense variants used as learning dataset [23,24].

**Figure 6:**
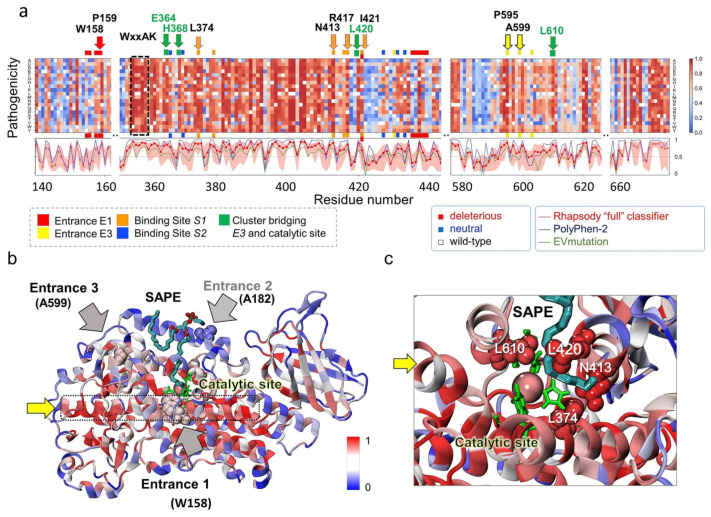
In silico saturation mutagenesis results for human 15LOX-2. (**a**) Pathogenicity probabilities for all substitutions, plotted as a function of residue number (abscissa) for all possible amino acid substitutions (ordinate). The probabilities are represented by a heatmap color-coded from blue (neutral) to red (deleterious). Colored horizontal bars (red, yellow, orange, and blue) along the upper abscissa denote the sites linked to specific functions (see labels under the map, Figure 2 and Table S2). Those distinguished by highly deleterious response to substitution are labeled. Black dashed box highlights the conserved WxxAK motif. The curves in the panel under the map indicate the sensitivity of a given residue to any mutation, as predicted by Rhapsody (red curve), EVmutation (green), and PolyPhen-2 (blue). Entrance 2 (on α2 helix) is broadly neutral to substitutions and not included in the heatmap. (**b**) Ribbon diagram of 15LOX-2/SAPE color-coded by pathogenicity probabilities (if mutated). The regions shown in space-filling representation and labeled (pointed by the black/grey arrows) are the entrances E1–E3. Catalytic residues are displayed in green and SAPE as cyan-red-blue sticks. (**c**) Close-up view of the catalytic site with bound substrate coordinated by four residues, L374, L420, L610, and N413, whose substitutions would be highly damaging to function. Yellow arrow points to the α12-14 scaffolding helix.

The results for residue segments of interest are presented as a heatmap in Figure 6a, color-coded from blue (neutral) to red (deleterious). The wild-type (WT) residues are shown in white. The ribbon diagram in panel b is also colored by the expected ‘pathogenicity’ from blue to red, mainly using the average values plotted under the heat map for each residue. The three curves therein represent the residue-averaged pathogenicity values predicted by Rhapsody (red), EVmutation [25] (green), and PolyPhen-2 [26] (blue). EVmutation takes rigorous account of residue (co)evolutionary properties and has proven to provide highly accurate results, while Polyphen-2 is broadly used for evaluating the effect of mutations due to its applicability in the absence of structural data or sufficiently large multiple-sequence-alignments. These curves provide a consolidated view of the sensitivity of 15LOX-2 amino acids to mutations, the peaks describing the sites that would be most resistant to substitutions in general.

As expected, substitutions at the catalytic residues H373, H378, H553, and I676, and at the WxxAK motif were deleterious, irrespective of the type of amino acid substitution. Of interest is, however, to see whether (or which of) the residues involved in mediating the interactions with the ligand (O_2_/NO^●^) or substrate (SAPE) are predicted to be critical to function. Our analysis in fact revealed that the residues labeled along the upper abscissa, written in boldface in Table S2, to be intolerant to mutations. These residues include P595 and A599 lining *E3*, W158-P159 and I421 at *E1*, and N413, R417, L421, and L374 at site *S1*. Among them we note that some play a dual role of coordinating the substrate SAPE too (L374, N413, L420, and L610; see Figure 6c). In contrast, the α2-helix residues A177-G199 (*E2* residues and binding interface for PEBP1) are found to be tolerant to mutations (Figure 6b), as well as the surface-exposed G189, even though it participates in 70% of the interactions with SAPE.

Residues located along the helix α12-14 (K350-Q391) are particularly sensitive to substitutions. Examination of the structure shows that this region, composed of a long helix (with disruptions at two positions, hence the labeling as α12, α13, and α14) spans the entire structure at the center, making contacts with both *E1* and *E3* residues and lining the catalytic pocket (Figure 6b, dotted black box; see also Supplementary Figure S1). Its scaffolding role and multiple contacts appear to be critical for maintaining the stability and functionality of the enzyme.

### 2.9. Identification of Nitrosylated PE Species in Cells Treated with NO^●^ Donors

**Figure 7:**
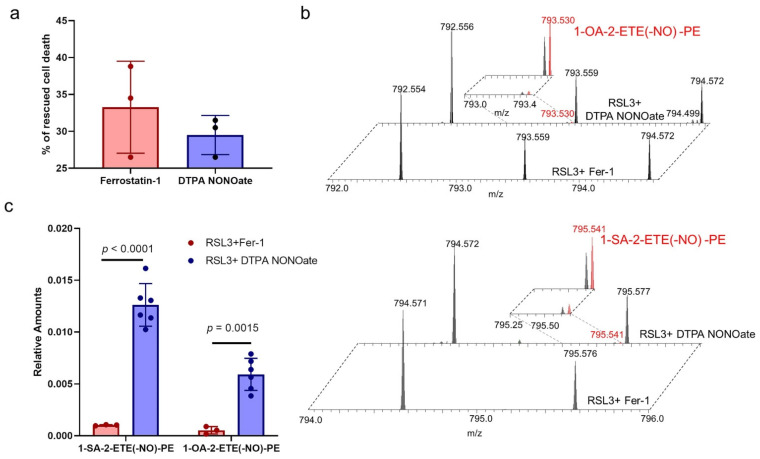
Formation of nitrosylated PE during the inhibition of ferroptosis by NO^●^ (**a**) Bar graph showing the decrease in cell death upon addition of ferroptosis inhibitor Ferrostatin-1 and DTPA NONOate in RAW 264.7 M2 macrophages treated with RSL3. Data are mean ± SD, *n* = 3 and no statistical significance was observed. (**b**) Mass spectrum showing the presence of two nitrosylated PE species, 1-OA-2-ETE(-NO)-PE (top panel) and 1-SA-2-ETE(-NO)-PE (bottom panel) in RAW 264.7 M2 macrophages treated with RSL3+Fer-1 (bottom section) and RSL3+DTPA NONOate (top section). Inset shows the magnified spectrum. **(c)** Quantities of 1-OA-2-ETE(-NO)-PE and 1-SA-2-ETE(-NO)-PE in RAW 264.7 M2 macrophages treated with RSL3+Fer-1 and RSL3+DTPA NONOate. Data are mean ± SD, *n* = 3 for RSL3+Fer-1 cells and 6 for RSL3+DTPA NONOate cells. *p* values are calculated using two-way ANOVA followed by Sidak post-hoc test.

Encouraged by these results, we next examined whether the nitrosylated PE products are formed in cells in which ferroptosis is inhibited by NO^●^. We had previously shown that RAW 264.7 M2 macrophages are susceptible to ferroptosis when their phospholipid hydroperoxide-specific glutathione peroxidase is inhibited by RSL3 [17]. In these cells, ferroptosis induced by RSL3 is suppressed by two inhibitors, Ferrostatin-1 and DTPA NONOate. Ferrostatin-1 suppresses ferroptosis by inhibiting the 15LOX-2/PEBP1 complex and through radical trapping antioxidant action. DTPA NONOate is a donor of NO^●^ [3]. We had earlier shown that rescue from ferroptosis by DTPA NONOate is associated with a reduction in HPETE-PE contents [17]. Both Fer-1 and DTPA NONOate rescued from RSL3 induced ferroptosis with approximately similar effectiveness (Figure 7a). We identified two nitrosylated PE products 1-SA-2-ETE(-NO)-PE and 1-OA-2-ETE(-NO)-PE with m/z values of 795.541 and 793.530, respectively (Figure 7b). The precursors for these nitrosylated lipids, 1-SA-ETE-PE and 1-OA-ETE-PE, are the two most abundant ETE-containing PE species in cells. There was about 10-fold excess of these nitosylated PE species in cells treated with DTPA NONOate compared to Fer-1 treated cells (Figure 7c). Though these amounts are very low and we do not have an appropriate standard to quantify these nitrosylated PE species, our findings point to the presence of NO^●^ in the close proximity of carbon-centered radicals formed by lipoxygenase.

## 3. Discussion

Among different aspects of ferroptosis, its physiological regulation by mechanisms other than the well-established effect of GPX4, attracted particular attention in recent years [27]. Among the most recent developments is a demonstration of iNOS/NO^●^ potency to directly control ferroptosis in macrophages and microglia, and distantly in several neighboring (e.g., epithelial) cells [13]. Interest in the role of NO^●^ in ferroptosis further increased by the possibility of using NO^●^ donors for attenuating, suppressing, or delaying ferroptotic death [13]. Although several mechanisms, including direct reaction of NO^●^ with lipid radical intermediates (L^●^, LO^●^, LO^●^_2_), have been considered [19], the exact nature of NO^●^’s inhibitory effect remained elusive.

Given the reported participation of 15LOX-2/PEBP1 complex in the generation of 15HpETE-PE that serve as a pro-apoptotic agent, NO^●^’s potential regulation of this catalytic complex has emerged as an important consideration. Our experiments in a model biochemical system with purified recombinant proteins directly demonstrate the ability of NO^●^ to inhibit the catalysis of ETE-PE oxidation to 15HpETE-PE by the complex but not by 15LOX-2 alone. This suggests that structural features specific to the 15LOX-2/PEBP1 complex could account for this inhibitory effect. Given that O_2_ is delivered to the 15LOX-2 catalytic site via a channel regulated by the binding of the oxidizable substrate, the role of structural changes in the 15LOX-2/PEBP1/SAPE in controlling O_2_ delivery and possible competition with NO^●^ became and even more fascinating yet unsolved puzzle. The present LC-MS data showing nitrosylation of ETE-PE, i.e., direct interaction of NO^●^ with a carbon-centered radical, rather than with the peroxyl radical intermediate, LO2^●^, provide further evidence for the direct interference of NO^●^.

The lipidomics experiments showed that the 15LOX-2/PEBP1 complex produced greater amount of the nitrosylated product (NO^●^-SAPE) than 15LOX-2 alone suggesting that a larger number of NO^●^ molecules were able to enter the catalytic pocket of PEBP1-bound 15LOX-2 compared to free 15LOX-2. In accord with these observations, our simulations showed the higher propensity of NO^●^ molecules to access the catalytic site in the presence of PEBP1 bound to 15LOX-2. In contrast, in the absence of PEBP1 and at small concentration of NO^●^, NO^●^ were attracted to the sn-1 tail of SAPE or grouped in a new cavity formed after loop L172-A179 reorganization in 15LOX-2 alone simulations. The experimentally observed formation of nitrosylated-ETE-PE catalyzed (albeit at lower levels) by 15LOX-2 alone [28], is possible due to presence of NO^●^ at the catalytic site. The in silico studies at similar concentrations of both molecules, could not detect any NO^●^ at the catalytic site. However, an increase in the number of NO^●^ molecules in the MD system lead to a few NO^●^ accessing the active site, explaining the nitrosylated-ETE-PE formation in 15LOX-2 alone. We also note that the turnover rate for 15LOX-2 is rather slow (~8.5–25/s). Thus, the current simulations mainly provide insights into the mechanistic aspects of NO^●^ actions, rather than a quantitative description of the overall kinetics.

Concentrations of both gases in physiological conditions may vary. Several studies indicate that the intra-cellular concentrations of O_2_ may be as high as 30 µM and the NO^•^ concentration can only go up to 5 µM [29]. In aerobically incubated cell culture, O_2_ can go up to 200 µM whereas NO^•^ concentration can be manipulated within the wide range dependently on the type of the NO^•^ donors added [30,31]. These concentrations may differ significantly within the micro-environment of 15LOX-2/PEBP1 complex. O_2_ is the substrate for many biologically relevant systems, such as cellular respiration [32], NADPH oxidase [33], dehydrogenases of the TCA cycle [34], while NO^•^ can be consumed in nitroxygenation and S-nitrosylation reactions of proteins and their thiols [35] and avidly react with O_2_^•^ to yield peroxynitrite [36]. Furthermore, the ratios of O_2_/NO^•^ may vary significantly dependently on the cell types. For example, in macrophages polarized to M1 phenotype, high expression of iNOS leads to a sharp increase in the NO^•^ production [37]. At the same time, NADPH oxidase consumes O_2_ to generate O_2_^•^ hence depleting O_2_ required for the 15LOX-catalyzed reactions [38]. Notably, iNOS generated NO^•^ can diffuse intra- and extra-cellularly to reach high levels sufficient for quenching the production of pro-ferroptotic signals by the 15LOX-2/PEBP1 complexes [13] (Dar et al., Manuscript under revision). In contrast M2 macrophages express negligible amounts of iNOS and NO^•^ likely resulting in preponderance of O_2_ vs. NO^•^ amounts [39]. Evidently, even more dramatic variations in intracellular contents of NO^•^ and O_2_ may occur in disease conditions related to inflammation and sepsis [40,41,42].

Our simulations unambiguously showed that this effect, occurring in the presence of PEBP1 is indeed due to the ability of NO^●^ molecules to bind and diffuse to the catalytic site of 15LOX-2, favored by a conformational change in 15LOX-2 induced upon complexation with PEBP1. The 15LOX-2/PEBP1/SAPE simulations revealed that the accessibility paths of both O_2_ and NO^●^ are similar, and NO^●^ often out-competes O_2_ and occupies the catalytic site in the 15LOX-2/PEBP1 complex, leading to the production of the nitrosylated product, NO^●^-SAPE observed in the biochemical and in vitro experiments. Notably, during free AA peroxidation, the NO^●^ molecules get access to the catalytic pocket. This suggests that at low concentration of NO^●^ molecules the exposed sn-1-acyl chain of SAPE in 15LOX-2 alone sequesters NO^●^ thereby preventing its access to the catalytic site. The lipidomics data further showed that lipid peroxidation by 15LOX-2 alone (ETE oxidation into 15-HpETE) was not altered significantly by NO^●^, in line with the lower accessibility to the 15LOX-2 catalytic site (in the absence of PEBP1) shown in MD simulations.

Finally, our work provides a detailed mechanistic description of the interactions of O_2_/NO^●^ molecules with 15LOX-2 residues during their journey towards the catalytic site. There are two available entrances to the catalytic cavity. The first (*E1*) is near Y154-W158 loop the flexibility of which is presumably limited by a proline, P159, highly resistant to substitutions. The second (*E2*) is near S430, S573, P595, A599, and L603. Both entrances provide access to the catalytic site through intra-protein channels that mediate the translocation of the small molecules. Our analysis highlighted the importance of L374, N413, L420, I421, and L610 in enabling efficient communication with the catalytic site. Detailed analysis of the substrate interaction site with 15LOX-2 and PEBP1 at the most favorable geometric positioning for lipids peroxidation also revealed crucial interactions with selected residues (L374, L420, L610, and N413) that formed a substrate-binding epitope.

Comparison of the critical residues with prior observations made for lipoxygenase family variants lends support to the functional significance of several sites identified here. For example, loss of 15LOX-2 activity has been observed in the variant A416D [43], a residue taking part in the binding site *S1* and in the cluster of hydrophobic residues that connect the entrance *E3* to the catalytic site. Likewise, the mutation N426M in 5LOX, which is the homologous counterpart of the site *S2* T431 in 15LOX-2, gives rise to a loss of activity when associated with F360W and A425I [44]. Likewise, the mutation A417A in LOX12 (counterpart of 15LOX-2 S430 at *E3*) reduced catalytic activity and altered the stereo-selectivity of the oxygenation reaction [45]. It is also worth noting that many of these identified residues are highly conserved across lipoxygenase family members as indicated in Supplementary Table S2.

The overall analysis identified not only key sites, but their intricate couplings to enable the catalytic activity of 15LOX-2 complexed with PEBP1. In the presence of NO^●^, the enzymatic machinery is still in place, but it cannot effectively produce HpETE-PE. Even though the direct reaction of NO^●^ with the ETE-PE carbon-centered intermediate takes place, albeit at a very low level, our analysis strongly suggests that the observed suppression of ETE-PE peroxidation by NO^●^ in the 15LOX-2/PEBP1 complex is mostly due to the occupancy of the O_2_-binding site or channeling path by NO^●^, and a competition between the two radicals (O_2_ and NO^●^) thus resulting in the reduced, if at low physiological NO^●^ amounts not completely abrogated, ETE-PE peroxidation. Interestingly, our highly sensitive redox lipidomics analysis did not reveal the formation of nitroxygenated ETE-PE derivatives. This suggests that the direct chemical reaction of NO^●^ with O_2_—very effective in gas and liquid phases [46]—is strongly suppressed within the structural confinements of the channel. It remains to be seen if this type of interference of NO^●^ to repress the peroxidation of free and esterified PUFAs by 15LOX-2/PEBP1 could be exploited in designing anti-ferroptotic therapies.

## 4. Materials and Methods

### 4.1. Molecular Dynamics (MD) Simulations

Conventional full-atomic MD simulations [47,48,49] were performed for human 15LOX-2 (PDB id: 4NRE [20]) and 15LOX-2/PEBP1 complex (PDB ids: 4NRE and 1BEH [50]), with a bound substrate (SAPE), in the presence of randomly distributed nitric oxide and oxygen molecules with different ratios, such as 1:1 (five of each), 1:3, and 3:1. Multiple MD runs (see Supplementary Table S1) of 150 ns with different initial spatial distributions of NO^●^ and O_2_ molecules were performed for each structure (15LOX-2 and its complex with PEBP1) using the NAMD [51] software with the CHARMM [52] force field and 2 fs time steps. The proteins were solvated with explicit water models (TIP3P [53]) at physiological salt concentrations. Docking simulations generated structural models for the 15LOX-2/PEBP1 complex, using the protocols described previously [3]. The binding site and pose of SAPE were predicted using SMINA [54] ligand-protein docking package derived from AutoDock Vina [55]. CHARMM force field parameters for NO^●^, O_2_, and covalently bonded Fe^3+^ were created based on bound O_2_ and heme group using Gaussian [56] package. Prior to productive runs, the following protocol was adopted: 0.2 ns of water equilibration, 10,000 steps of minimization, 0.35 ns of heating from 0 to 300 K, and 0.15 ns equilibration of the whole system. Simulations were performed with a cutoff of 12 Å for non-bonded interactions and Langevin piston algorithm to maintain the temperature at 300K and pressure at 1 atm. We used VMD [57] for visualization and ProDy [58,59,60] for trajectory analysis with in-house scripts. CAVER [61] 3.0 with PyMOL Molecular Graphics System, Version 1.8, Schrödinger, LLC was used to represent and display cavities and interior surfaces.

### 4.2. Rhapsody and in Silico Saturation Mutagenesis Analysis

The Rhapsody tool [24] was used for automated scanning of all residue substitutions in 15LOX-2 to predict the functional consequences of single amino acid variants (SAV). A random forest-based classifier was trained on an integrated dataset of 20,854 missense mutations functionally characterized to date. For each training sample, eight features incorporating the effects of structural dynamics and sequence-based (co)evolution properties were calculated using ProDy [58], Evol [58], and PolyPhen-2 [62]. The 15LOX-2 structure (PDB id: 4NRE [20]) was used as input. Residue-averaged scores evaluated by Rhapsody, PolyPhen-2, and EVmutation [25] were examined for consolidation of the results.

### 4.3. Lipoxygenase Assay

Human recombinant 15LOX-2 and PEBP1 were recombinantly expressed an purified as described previously [3,63]. 15LOX-2 was pre-activated with 13-hydroperoxy-octadecadienoic acid (HpODE) (5 μM) for 30 min at 37 °C. The pre-activated 15LOX-2 (3 pmols) was added to a reaction mixture containing 100 μM lipid in Tris-HCl (50 mM, pH7.4), 25 µM PAPA NONOAte and DTPA (100 μM) for a total volume of 50 μL. DTPA was added 15 min prior to the start of the reaction. For reactions with the 15LOX-2/PEBP1 complex, equal quantity of PEBP1 and 15LOX-2 were mixed prior to pre-activation. The reaction mixture was incubated on a shaker mixer at 37 °C for 30 min. For time course, the reaction was allowed to continue up to a specific time. The reaction was stopped with addition of 9 volumes (450 μL) of 100% acetonitrile, and the samples were centrifuged at 10,000× *g* for 15 min at 4 °C. Then, 20 μL of the supernatant was transferred to an auto sampler vial, and 5 μL was injected into the LC-MS/MS system. LC-MS/MS analysis was performed as described previously.

### 4.4. Cell Culture

RAW 264.7 cells were obtained from the American Type Culture Collection (ATCC). Cultured at 37 °C and 5% CO_2_ in DMEM or RPMI (ATCC) supplemented with 10% heat-inactivated fetal calf serum (FCS; Sigma–Aldrich, St. Louis, MO, USA) and 50 U ml^−1^ penicillin–streptomycin (Thermo Fisher Scientific, Waltham, MA, USA). RAW 264.7 macrophages were polarized by to M2 stage by incubating in DMEM containing 10% FBS, 50 U mL^−1^ penicillin–streptomycin, and IL-4 (20 ng mL^−1^) for 48 h. For ferroptotis experiments, cells were incubated with RSL3 (0.5 µM), 2 µM), +Fer-1 (~400 nM), or ±DPTA NONOate (25 µM), for 5 h. Cell death was determined by flow cytometry.

### 4.5. LC–MS Analysis of Phospholipids

MS analysis of phospholipids was performed on an Orbitrap mass spectrometer (Thermo Fisher). Phospholipids were separated on a normal phase column (Luna 3 µm Silica (2) 100 Å, 150 × 2.0 mm, Phenomenex, Torrance, CA, USA) at a flow rate of 0.2 mL min^−1^ on a Dionex Ultimate 3000 HPLC system (Dionex, Idstein, Germany). The column was maintained at 35 °C. Analysis was performed using gradient solvents (A and B) containing 10 mM ammonium acetate. Solvent A contained propanol:hexane:water (285:215:5, vol/vol/vol) and solvent B contained propanol:hexane:water (285:215:40, vol/vol/vol). All solvents were LC–MS grade. The column was eluted for 0–23 min with a linear gradient of 10–32% B; 23–32 min using a linear gradient of 32–65% B; 32–35 min with a linear gradient of 65–100% B; 35–62 min held at 100% B; 62–64 min with a linear gradient from 100% to 10% B followed by and equilibration from 64 to 80 min at 10% B. The instrument was operated with the electrospray ionization probe in negative polarity mode. Data was analyzed using Quan Browser of xcalibur software (Thermo Fisher).

## Data Availability

Data and codes generated during the study and included in this article are available from the corresponding authors upon request.

## Supplementary Materials

The following are available online at https://www.mdpi.com/1422-0067/22/10/5253/s1, Figure S1: Location of the two entrances E1 and E2 of 15LOX-2 that enable access of O_2_ and NO^●^ to the catalytic site, Figure S2: Secondary structure of 15LOX-2, Figure S3: Additional pores or tunnels leading to the catalytic site, Figure S4: Close-up view of contacts between 15LOX-2 residues and O_2_/NO^●^ molecules, Figure S5: Same results as Figure 4a–b, reproduced by an independent run, Figure S6: 15LOX-2/SAPE complex and its interactions with O_2_ and NO^●^ molecules observed in MD simulations with higher concentration of NO^●^ molecules, Figure S7: 15LOX-2/AA complex and its interactions with O_2_ and NO^●^ molecules observed in MD simulations, Table S1: Summary of the simulated systems, compositions, and durations, Table S2: Key residues in 15LOX-2 that play a role in regulation of lipid peroxidation, Movie 1: Competition between NO^●^ and O_2_ molecules near the SAPE at the catalytic site of 15LOX-2 complexed with PEBP1.
