# Supplementary material for: NO● Represses the Oxygenation of Arachidonoyl PE by 15LOX/PEBP1: Mechanism and Role in Ferroptosis
Source: Int J Mol Sci. 2021 May 17;22(10):5253. doi: 10.3390/ijms22105253 (PMC8156958; doi:10.3390/ijms22105253)
Supplement: Supplementary file 1 [file ijms-22-05253-s001.zip › 3_IJMS_Supplementary_Material.pdf]

Supplementary Material for

NO• represses the oxygenation of arachidonoyl PE by 15LOX/PEBP1: Mechanism and role in ferroptosis

Karolina Mikulska-Ruminska[1,2*], Tamil S. Anthonymuthu[4], Anastasia Levkina[3, 9], Indira H. Shrivastava[1,3], Alexandr A. Kapralov[3], Hulya Bayır[3,4], Valerian E. Kagan[3,5,6,7,8†*], Ivet Bahar[1†*]

[1] Department of Computational and Systems Biology, School of Medicine, University of Pittsburgh, Pennsylvania 15260, USA
[2] Institute of Physics, Faculty of Physics, Astronomy and Informatics, Nicolaus Copernicus University in Toruń, Grudziadzka 5, 87-100 Toruń, Poland
[3] Department of Environmental and Occupational Health and Center for Free Radical and Antioxidant Health University of Pittsburgh, Pittsburgh, Pennsylvania 15260, United States
[4] Department of Critical Care Medicine, Safar Center for Resuscitation Research, Children's Neuroscience Institute, Children's Hospital of Pittsburgh, University of Pittsburgh, Pittsburgh, Pennsylvania 15260, USA
[5] Department of Radiation Oncology, [6]Department of Chemistry, and [7]Department of Pharmacology and Chemical Biology, University of Pittsburgh, Pittsburgh, Pennsylvania 15260, USA
[8] Institute of Regenerative Medicine, IM Sechenov Moscow State Medical University, Moscow 119048, Russia

† - I.B. and V.E.K. contributed equally to this work
* Correspondence: karolamik@fizyka.umk.pl (K.M-R); kagan@pitt.edu (V.E.K.); bahar@pitt.edu (I.B.)

Supplementary Figures

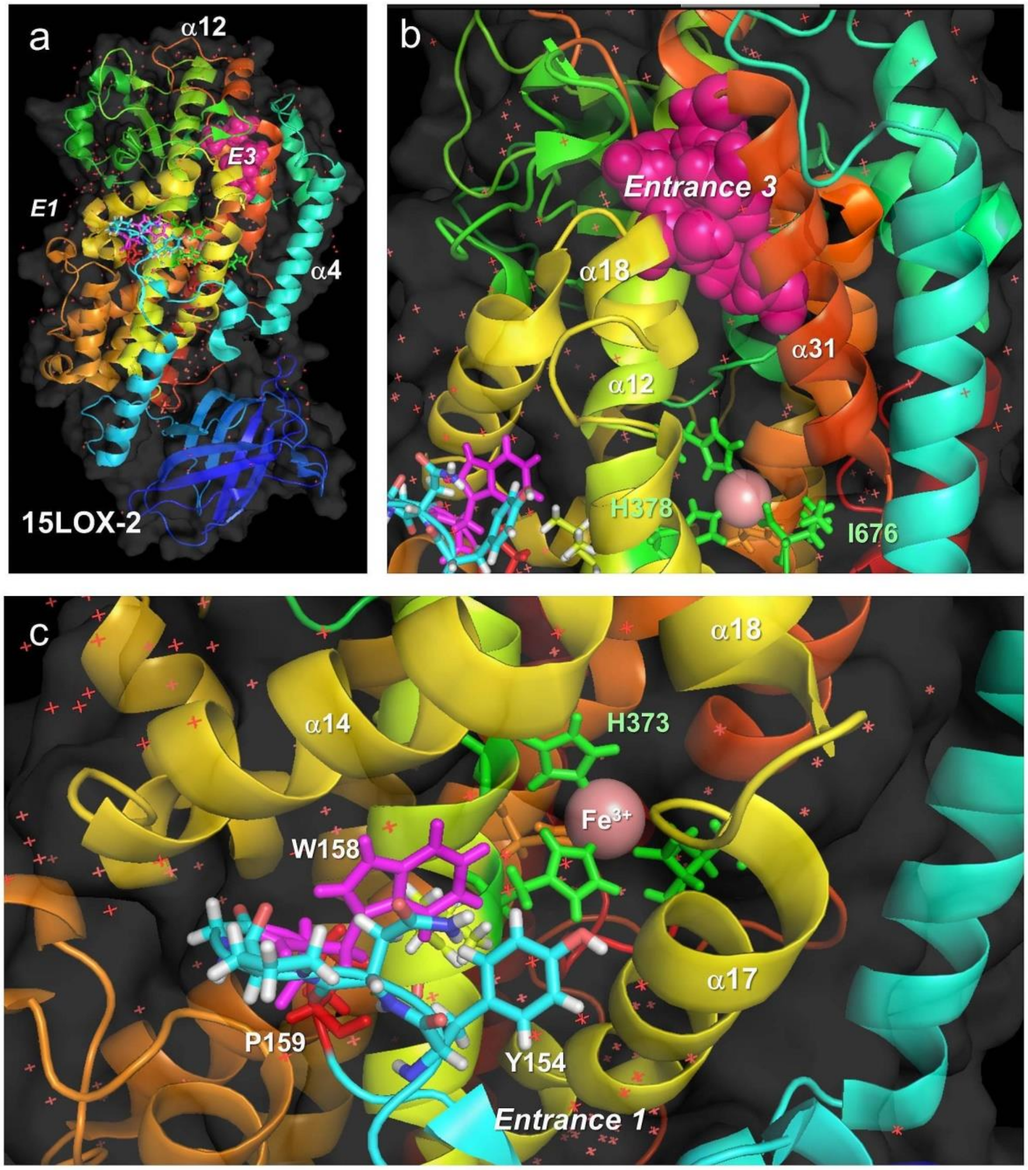

Figure S1. Location of the two entrances *E1* and *E2* of 15LOX-2 that enable access of O_2 and NO• to the catalytic site. (a) Overall structure (based on the structure resolved [1] for 15LOX (PDB: 4NRE), color coded by chain from *blue* (N-terminus) to *red* (C-terminus). The protein is rotated by 90° with respect to the view shown in Figure 2a to enable a clearer view of the two entrances. The first helical portion (α12; *green*) of the long helix α12-14 that spans the overall structure is labeled. Its other portions (*green to yellow*) making contacts with either entrance and lining the catalytic site are labeled in other panels. **(b)** Entrance 3 residues (S430, S573, P595, A599 and V603), shown in *hot red space-filling representation* located between helices α18 and α31

(see **Figure S2**), providing access to the catalytic residues shown in *green sticks*. Note that one of the catalytic residues (H553, colored *orange*) is not visible from this perspective, being located behind the Fe^{3+} ion (*pink sphere*); **(c)** Close-up view of the loop residues Y154-P159 (shown in *sticks*) that form the entrance 1 (*E1*). Note that Y154 (*cyan*, with side chain hydroxyl O in *red*) and W158 (*magenta*) may play a gating role upon rotational isomerization. P159 does not directly participate in the porous region but is a highly conserved residues that presumably provides a scaffold for restraining the loop motion. See also **Figure S3**.

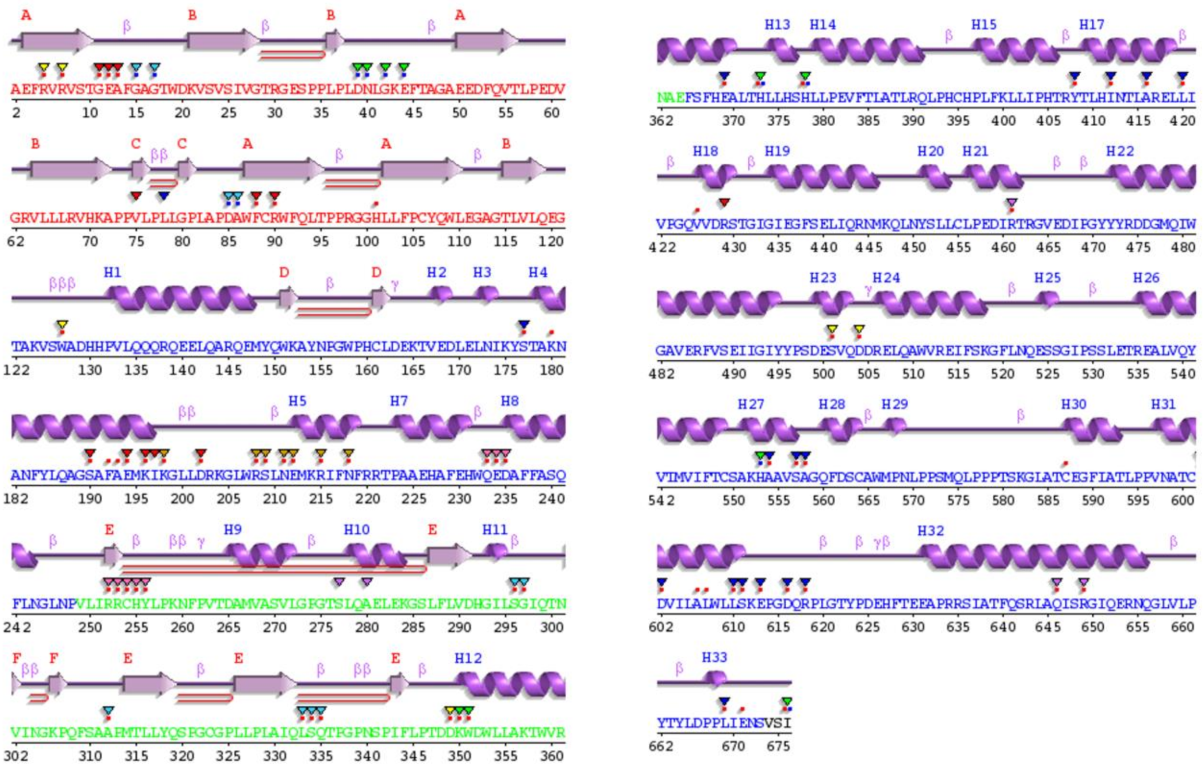

Figure S2. Secondary structure of 15LOX-2. The diagram displays the ranges of amino acids (from PDBsum [2] for 4NRE) corresponding to the structural elements referred to in the text, figures, and **Table S2**. Residue numbers corresponding to the N-terminal domain, composed of β-strands, are written in *red*.

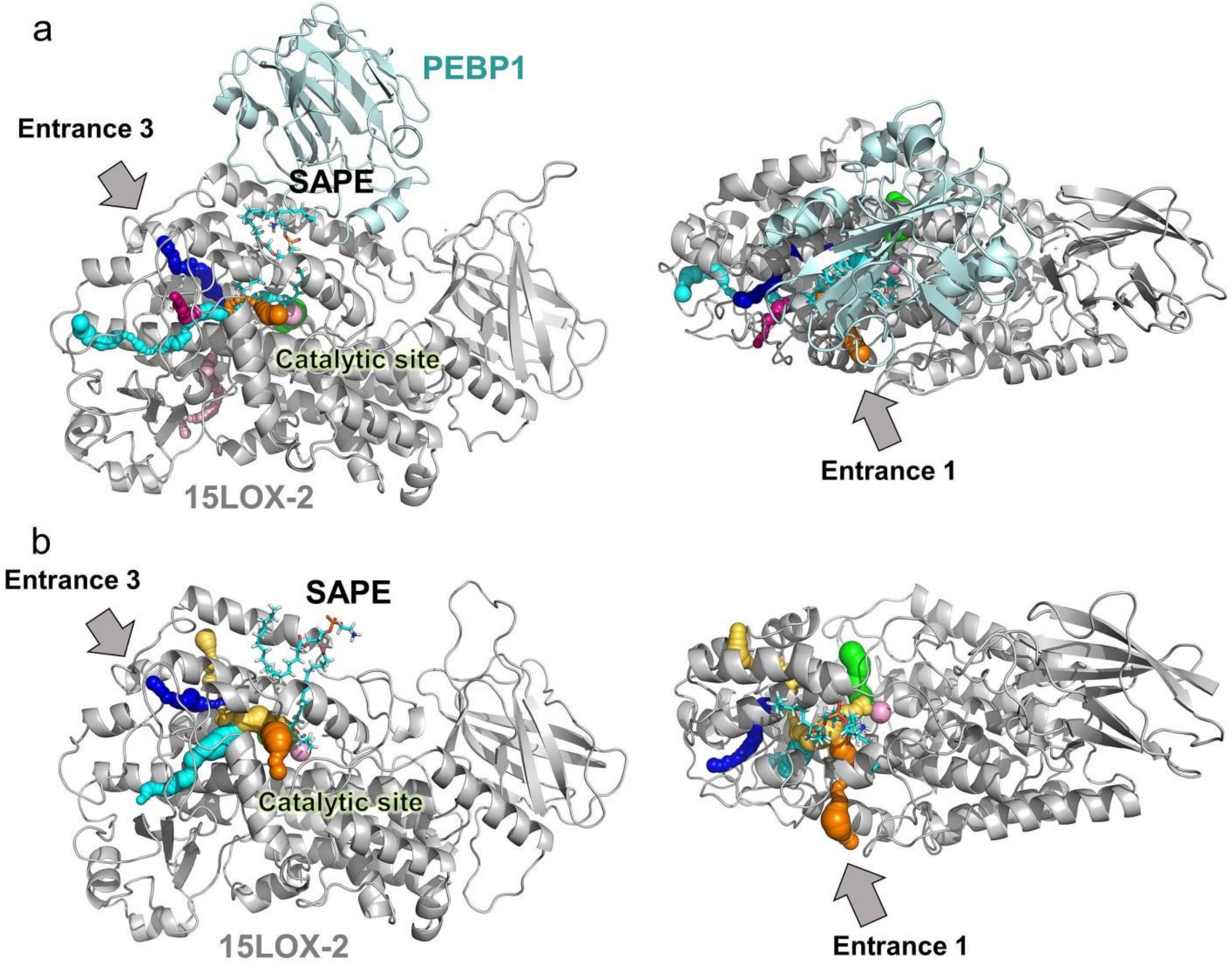

Figure S3. **Additional pores/tunnels leading to the catalytic site.** Cavities and interior surfaces that lead to the catalytic site of 15LOX-2 were detected in **(a)** 15LOX-2/PEBP1/SAPE and **(b)** 15LOX-2/AA using Caver [3]. These pores/tunnels are shown in different colors (*cyan, orange, blue, green* and *yellow*), surface representation. SAPE atoms are shown as *sticks* with carbons in *cyan* and oxygens in *red*. Entrances 1 and 3 are pointed by *black arrows*, respectively. Diagrams on the *right* are the views from *top*.

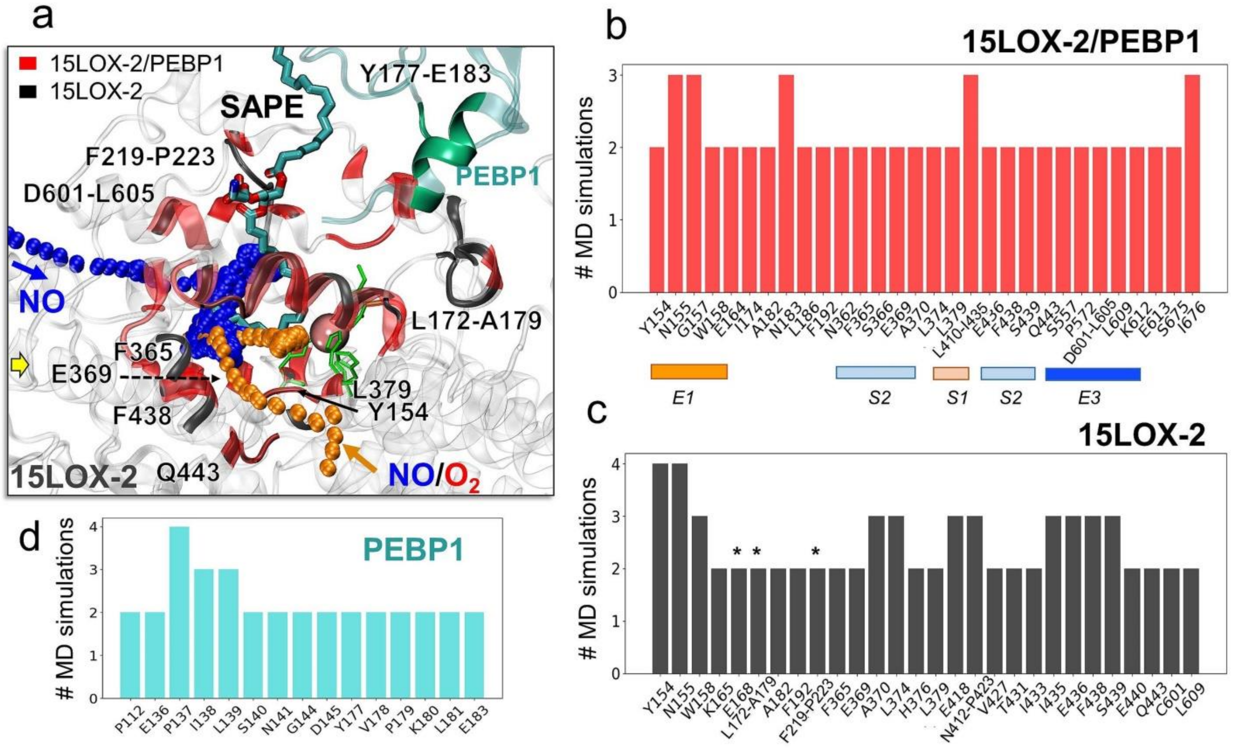

Figure S4. Close-up view of contacts between 15LOX-2 residues and O_2/NO• molecules. (a) The 15LOX segments that exhibit the most frequent interactions with NO• and O_2 molecules are displayed in *red*/*green* for 15LOX-2/PEBP1/SAPE and in *black* for 15LOX-2/SAPE. Catalytic residues are displayed in *green sticks*. O_2 and NO• pathways to the catalytic site through Entrance 1 (*orange*) and Entrance 3 (*blue*) are shown. *Yellow arrow* points to α12-14. **(b-c)** Histograms of 15LOX-2 residues which make contacts with O_2/NO• in the presence **(b)** or absence **(c)** of PEBP1. The horizontal bars between the panels indicate the residues located at the entrances *E1* and *E3*, or sites *S1 and* S2. An O_2 or NO• molecule is assumed to make a contact with 15LOX-2 if it stays within 3.5 Å of any 15LOX-2 atom for at least 3 ns during the course of a 150 ns run. Residues observed to make contacts only in one run are not displayed. *Black stars* in panel c denote the regions that are not occupied by NO• and O_2 in the presence of PEBP1. **(d)** PEBP1 residues making extended contacts with NO• and O_2 in at least two independent runs.

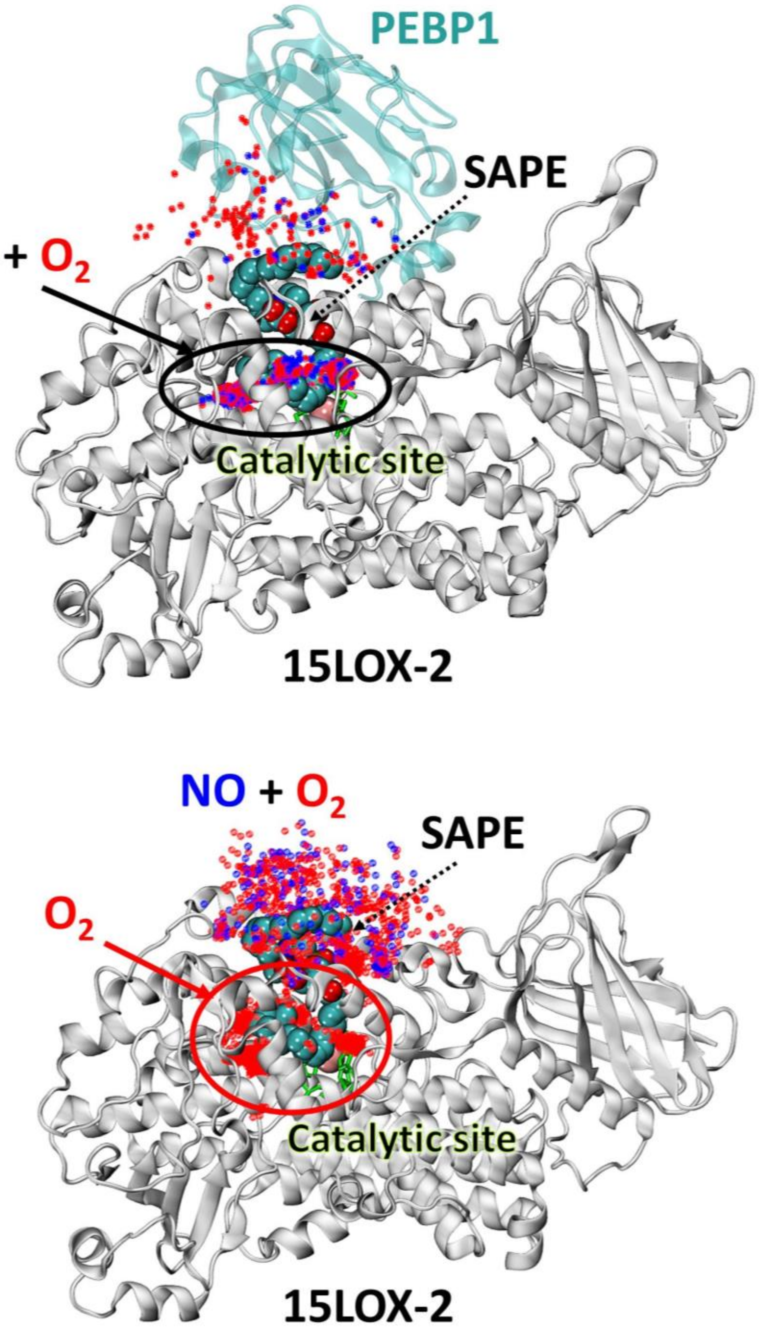

Figure S5. Same results as Figure 4a-b, reproduced by an independent run. The repeated pattern shows the robustness of the preferential binding positions of O_2 and $NO^•$ in the presence (*top panel*) and absence (*bottom panel*) of PEBP1 complexation.

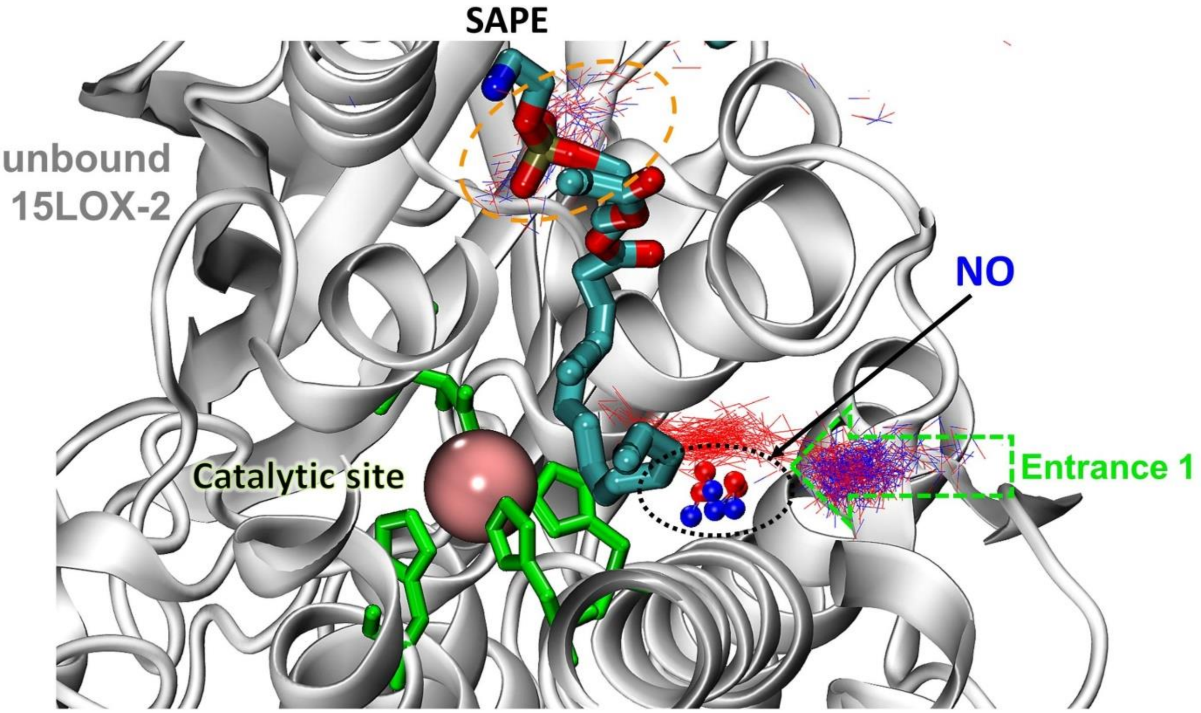

Figure S6. 15LOX-2/SAPE complex and its interactions with O_2 and NO• molecules observed in MD simulations with higher concentration of NO• molecules. The positions of the O_2/NO• molecules within 7 Å of SAPE observed in multiple snapshots are shown in *red/blue dots*. Both small molecules are observed to visit the catalytic site. The cloud of small dots shows where and how long (more dots) O_2 and NO• travelled and/or remained bound during simulations. *Orange sphere* denote accumulation of NO• molecules near the loop L172-A179 (see also Figure 4c).

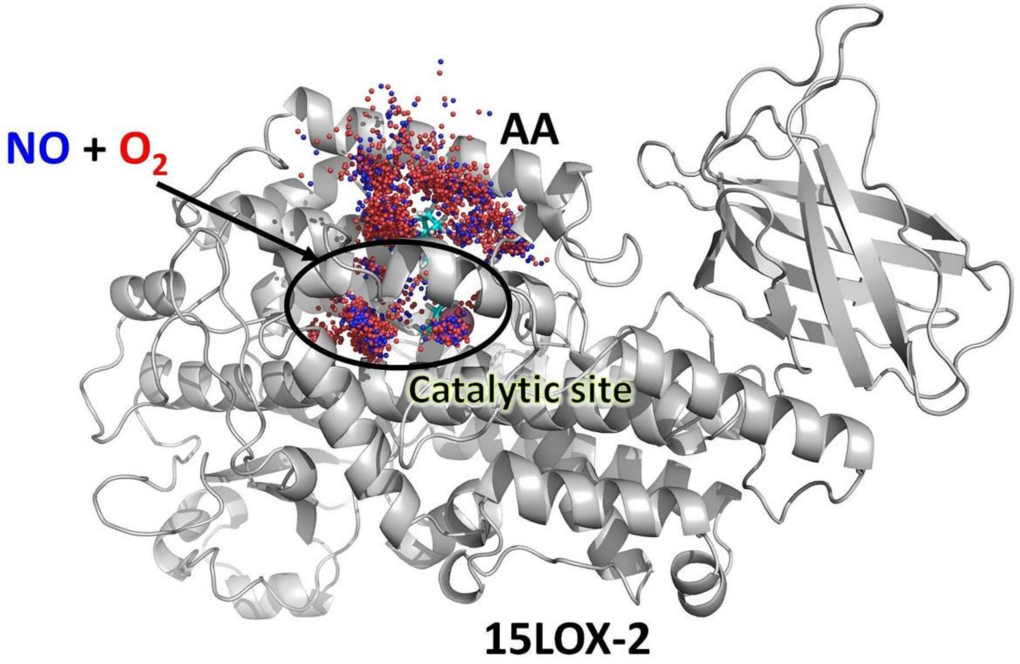

Figure S7. 15LOX-2/AA complex and its interactions with O_2 and NO• molecules observed in MD simulations. The positions of the O_2/NO• molecules within 7 Å of the substrate (AA or ETE) observed in multiple snapshots are shown in *red/blue dots*. Both small molecules are observed to visit the catalytic site.

Supplementary Tables

Table S1. Summary of the simulated systems, compositions, and durations.

Simulation system (substrate/ligands in addition to 15LOX-2)	# of H_2O molecules	#of O_2	# of NO molecules	Total # of Atoms	# of Runs	Total Time per run (ns)	Total simulation duration (ns)
+ AA+O_2	15 266	5	-	56 611	2	150	300
+ AA+NO+O_2	21 249	5	5	56 570	3	150	450
+ SAPE+O_2	15 251	5	-	56 643	2	150	300
+ SAPE+O_2+NO	15 235	5	5	56 606	2	150	300
+ PEBP1+SAPE+O_2	20 977	5	-	76 730	2	150	300
PEBP1+SAPE+O_2+NO	20 960	5	5	76 707	5	150	750
+ SAPE+O_2+NO	15 674	15	5	57 944	2	150	300
PEBP1+SAPE+O_2+NO	21 214	15	5	77 491	2	150	300
+ SAPE+O_2+NO	15 674	5	15	57 944	2	150	300
PEBP1+SAPE+O_2+NO	20 926	5	15	76 625	2	150	300

Table S2. **Key residues in 15LOX-2 that play a role in regulation of lipid peroxidation[1, 2].**

Role of residue	15LOX-2	15LOX-1	5LOX	LOX12
Catalytic residues	H378	H365	H373	H365
	H373	H360	H368	H360
	H553	H540	H551	H540
	I676	I662	I674	I663
Entrance 1 (*E1*) to a pore on 15LOX surface (Figure 1b), which permits O_2• (and NO•) to have access to the catalytic site though sites *S1* or *S2*	Y154	W144	W148	W144
	N155	K145	N149	K145
	G157	G147	G151	G147
	W158	L148	F152	L148
	I421	V408	I416	I408
	I435	G422	G430	G422
	F438	H425	H433	H425
	S439	V426	V434	V426
Binding site *S1* (Figure 1c) for small molecule binding after entry from *E1*	N413	N400	N408	N400
	A416	A403	A411	A403
	R417	R404	R412	R404
	L374	L361	L369	L361
	L379	L366	L374	L366
Binding site *S2* (Figure 1c) for small molecules (O_2• and NO•) entering through either *E1* or *E3*	I433	T420	T428	T420
	F365	F352	F360	F352
	T431	M418	N426	V418
	V427	F414	F422	F414
	E369	E356	Q364	E365
Entrance 3 (*E3*) residues (Figure 1d)	S573	T560	T571	T560
	P595	P581	P593	P582
	A599	Q585	R597	Q586
	V603	Q589	H603	Q590
	S430	I417	A425	A417
Cluster 1 of hydrophobic residues bridging between ***E3*** and catalytic site (exhibiting extended interactions with NO•/O_2•)	I216	I206	I211	I206
	I604	M590	L602	M591
	F561	L548	Y599	L548
	C564	Y551	C562	Y551
	A565	S552	S563	A552
Cluster 2 of hydrophobic residues bridging betweer and catalytic site	L607	T593	V605	S594
	L610	L596	L608	L597
	L420	L407	L415	L407
	V426	I413	L421	I413
	V427	F414	F422	F414
Cluster 3 of residues bridging between ***E3*** and catalytic site, also merging with site S2	H368	H355	H363	H355
	L570	A577	A568	A557
	L246	A236	C241	A236
	E364	D351	D359	D351
Residues which coordinate the sn-2 (ETE) chain of SAPE near the catalytic site of 15LOX (Figure 6c)	L610	L596	L608	L597
	L420	L407	L415	L407
	N413	N400	N408	N400
	L374	L361	L369	L361
Residues that exhibit frequent interactions with SAPE (Figure 5)	G189	A179	S183	K179
	A416	A403	A411	A403

	A606	I592	A604	I593
Binding of SAPE to 15LOX-2/PEBP1	Y185	E175	V179	E175
Binding of SAPE to 15LOX-2 (Figure 5)	N181	R171	G175	R171
Additional tunnel detected by Caver (Figure S1b, *green*)[3]	S557<br>E613<br>Q560<br>L610	H554<br>R599<br>Q547<br>L596	N555<br>F611<br>Q558<br>L608	N554<br>R600<br>Q547<br>L597
WxxAK motif	W353-K357	W340-K344	W348-K352	W340-K344

[1]In red are highly conserved residues; [2] Current analysis was performed for 15LOX-2. Three other family members are displayed for more information. [3]in addition to the catalytic residues H373, H553 and I676

References

1. Kobe, M. J.; Neau, D. B.; Mitchell, C. E.; Bartlett, S. G.; Newcomer, M. E., The structure of human 15-lipoxygenase-2 with a substrate mimic. *J. Biol. Chem.* **2014,** 289, (12), 8562-8569.
2. Laskowski, R. A., PDBsum: summaries and analyses of PDB structures. *Nuc. Aci. Res.* **2001,** 29, (1), 221-222.
3. Chovancova, E.; Pavelka, A.; Benes, P.; Strnad, O.; Brezovsky, J.; Kozlikova, B.; Gora, A.; Sustr, V.; Klvana, M.; Medek, P., CAVER 3.0: a tool for the analysis of transport pathways in dynamic protein structures. *PLoS Comput Biol* **2012,** 8, (10), e1002708.
